# Deficiency of TREK-1 potassium channel exacerbates blood-brain barrier damage and neuroinflammation after intracerebral hemorrhage in mice

**DOI:** 10.1186/s12974-019-1485-5

**Published:** 2019-05-09

**Authors:** Yongkang Fang, Yeye Tian, Qibao Huang, Yue Wan, Li Xu, Wei Wang, Dengji Pan, Suiqiang Zhu, Minjie Xie

**Affiliations:** 1 Department of Neurology, Tongji Hospital, Tongji Medical College, Huazhong University of Science and Technology, Wuhan, People’s Republic of China 430030; 2 College of medicine, Wuhan University of Science and Technology, Wuhan, 430081 People’s Republic of China; 3 Department of Neurology, The Third People’s Hospital of Hubei Province, Wuhan, People’s Republic of China 430030

**Keywords:** TREK-1, Intracerebral hemorrhage, Secondary injury, Inflammation, Blood-brain barrier

## Abstract

**Background:**

Intracerebral hemorrhage (ICH) is a devastating medical emergency with high mortality and severe neurological deficit. ICH-related poor outcomes are due to a combination of pathological processes that could be complicated by secondary insults. TWIK-related K+ channel 1 (TREK-1) is a two-pore-domain potassium channel that is highly expressed in the mammalian nervous system. Previous studies have shown that TREK-1 channels play important roles in various central nervous system diseases. However, its role in the secondary injuries after intracerebral hemorrhage remains unknown. In this study, we explored the function of TREK-1 in secondary blood-brain barrier injuries and neuroinflammation after intracerebral hemorrhage in mice.

**Methods:**

Adult male TREK-1^−/−^ mice and WT mice were subjected to a collagenase-induced ICH model. Immunostaining, western blot, and enzyme-linked immunosorbent assay were used to assess inflammatory infiltration and neuronal death. Blood-brain barrier compromise was assessed using electron microscopy and Evans Blue dye injection on days 1 and 3 after intracerebral hemorrhage. Magnetic resonance imaging and behavioral assessments were conducted to evaluate the neurologic damage and recovery after intracerebral hemorrhage.

**Results:**

Genetic deficiency of TREK-1 channel exacerbated blood-brain barrier impairment and promoted cerebral edema after intracerebral hemorrhage. Meanwhile, TREK-1 deficiency aggravated focal inflammatory featured by the increased recruitment of microglia and neutrophils, the enhanced secretion of proinflammatory factors interleukin-1 beta (IL-1β), tumor necrosis factor alpha (TNF-α), and cell adhesion molecules (CAMs). Furthermore, TREK-1 deficiency promoted neuronal injury and neurological impairment.

**Conclusions:**

These results establish the first in vivo evidence for the protective role of TREK-1 in blood-brain barrier injury and neuroinflammation after intracerebral hemorrhage. TREK-1 may thereby be harnessed to a potential therapeutical target for the treatment of intracerebral hemorrhage.

**Electronic supplementary material:**

The online version of this article (10.1186/s12974-019-1485-5) contains supplementary material, which is available to authorized users.

## Introduction

Intracerebral hemorrhage (ICH) is a devastating stroke subtype, associated with high mortality and severe neurologic impairment [1]. Pathological processes after ICH include the combined effects of primary injury and secondary injury [2]. The primary injury is featured by the mechanical tissue damage caused by initial hematoma. The secondary injuries usually include inflammatory cell infiltration, coagulation factors, neuronal death, and breakdown of the blood-brain barrier (BBB). Previous studies have demonstrated that BBB disruption is the hallmark of ICH-induced brain injury [3]. The increased BBB permeability contributes to the influx of leukocytes and cerebral edema formation. Neuroinflammation following ICH involves activation of microglia, recruitment of peripheral leukocytes, and secretion of proinflammatory factors. These inflammatory events may further induce BBB disruption and brain edema, which ultimately lead to neuronal death and neurological deterioration [2, 4, 5]. Therefore, a therapeutic strategy to maintain the BBB integrity and inhibit neuroinflammatory cascade will become a promising treatment of cerebral hemorrhage.

TWIK-related K^+^ channel 1 (TREK-1) potassium channel is a member of the newly discovered two-pore-domain background potassium (K2P) channels that are widely expressed in the central nervous system (CNS) [6, 7]. Through hyperpolarizing the membrane potential, these channels exert crucial roles in the normal function of the nervous system. The gating properties of TREK-1 are regulated by numerous physiological/pathological stimuli including membrane stretch, heat, intracellular acidosis, volatile anesthetics, and unsaturated fatty acids [8, 9]. Accumulating studies have demonstrated that TREK-1 plays a key role in the cellular mechanisms of neuroprotection, anesthesia, pain, depression, and spinal cord injury [10–14]. The polyunsaturated fatty is neuroprotective against global ischemia and kainate-induced epilepsy mediated by TREK-1 opening [12, 14–16]. In a mouse experimental autoimmune encephalomyelitis (EAE) model, pharmacological block or genetic knockout (KO) TREK-1 channels can lead to increased inflammatory infiltration and neurological impairment [17, 18]. However, the function of TREK-1 in the secondary injury after ICH remains unknown.

Previous studies have indicated that TREK-1 is extensively expressed in neurons, astrocytes and vascular endothelial cells that are indispensable parts of the BBB [6, 7]. TREK-1 has been shown to regulate inflammatory responses and BBB integrity in a mouse experimental autoimmune encephalomyelitis (EAE) model [18]. Here, we test whether TREK-1 could exert a protective effect in the secondary injury after ICH. Using a collagenase-induced ICH model, we sought to investigate the potential role of TREK-1 in the inflammation and BBB integrity after ICH. Our results show that genetic deletion of TREK-1 can increase the permeability of BBB, aggravate inflammatory infiltration, neurons apoptosis, and inhibit neurological functional recovery. These outcomes suggest that TREK-1 may be a promising therapeutical target to treat the secondary damage after ICH.

## Materials and methods

### Animals

All animal experiments were made under a protocol approved by the Committee on the Ethics of Animal Experiments and the Institutional Animal Care and Use Committee at Tongji Medical College, Huazhong University of Science and Technology. The TREK-1^−/−^ mice were generated as previously reported [14, 19]. TREK-1^−/−^ mice are healthy, fertile, and do not display any morphological abnormalities. All mice including WT C57BL/6 and TREK-1^−/−^ mice were maintained in the specific pathogen-free conditions in the animal facilities at Tongji Medical College. They were given access to a 12 h light/dark cycle in an 18~22 °C facility, with free access to food and water. A total of 213 adult male mice (8–10 weeks) including WT C57BL/6 mice and TREK-1^−/−^ mice were randomly assigned into four groups: the WT sham group, WT ICH group, KO sham group, and KO ICH group (Additional file 1: Table S1).

### ICH surgery

The collagenase-induced ICH model was established as described previously [20]. Briefly, mice were anesthetized with an intraperitoneal injection of 60 mg/kg ketamine and 10 mg/kg promethazine and fixed on a mouse stereotaxic apparatus. A 0.5-mm-diameter burr hole was drilled at the following coordinates relative to bregma: 0.5 mm anterior, 2.5 mm right lateral, 4 mm deep. Thereafter, 0.5 μl saline containing 0.075 U of collagenase VII-S (Sigma-Aldrich, St Louis, MO) was administered using a 1 μl microsyringe (Gauge, Shanghai, China) over a period of 2.5 min at a speed of 2 μl/min. The needle was kept for 10 min after injection, then the microsyringe was pulled out. The hole was subsequently sealed with bone wax, and the scalp was closed with sutures. Body temperature was maintained at 37 °C throughout the surgery using a heat lamp. The sham-operated group had similar procedures without infusion with collagenase. The mice died of anesthesia were excluded.

### Quantification of ICH volume

We quantified the ICH volume using Cavalieri’s method of morphometric volume [14]. Briefly, seven mice, respectively from the WT ICH and KO ICH group were chosen for the detection of ICH volume on days 1 and 3 after ICH surgery. Ten cryosections from each mouse with 10 mm thickness, 100 mm intervals apart through the ventral to the dorsal of hematoma were applied for hematoxylin-eosin (HE) staining. The ICH volume = (SUM hemorrhage area × distance between sections)−(epicenter hemorrhage area × section thickness). The hematoma area was quantified using ImageJ software (National Institutes of Health, Bethesda, MD, USA). All measurements were performed blindly.

### Brain water content measurement

Brain edema was assessed by the wet/dry method as described previously [21]. Briefly, mice were euthanized on days 1, 3, and 7 after ICH. The brains were removed and immediately separated into hemorrhage hemispheres, contralateral hemispheres, and cerebellums. Then, each part was weighed using an electronic analytical balance (Sartorius BS 210 S, Gottingen, Germany) to obtain the wet weight (WW). The cerebellum was used as an internal control. Then the tissues were dried at 110 °C for 24 h to get the dry weight (DW). The water content was calculated as a percentage of the wet weight: (WW−DW)/(WW) × 100%.

### Tissue preparation

After anesthetizing by an overdose of ketamine/xylazine, mice were sacrificed and trans-cardiac perfused with 4% paraformaldehyde on days 1 and 3 post-ICH. The brains were removed, stored in 4% paraformaldehyde at 4 °C overnight, and then were transferred into 30% sucrose at 4 °C for 3 days. The brains were sectioned with a cryostat at 10 μm thickness and stored at − 20 °C for further experiments and analysis. For immunofluorescent staining and western blot analysis, tissue within 1.5 mm of the hematoma was identified as perihematoma tissue.

### Immunofluorescence and terminal-deoxynucleotidyl TUNEL staining

A series of sections were washed in phosphate buffer solution (PBS), blocked in 10% bovine serum albumin (BSA) for 1 h at 25 °C. Then, these slices were incubated at 4 °C for 12–16 h with primary antibodies (Additional file 1: Table S2) including rabbit anti-TREK-1 (1:200; Alomone Lab, Jerusalem, Israel), goat anti-mouse glial fibrillary acidic protein (GFAP) (1:200; Abcam Shanghai, China), rabbit anti-Iba1 (1:200; Wako, Osaka, Japan), mouse anti-MPO (1:100; Santa Cruz, Biotechnology, TX, USA), rat-anti mouse CD31 (1:100, BD Bioscience , New Jersey, USA), rabbit-anti mouse ICAM1 (1:100, Proteintech, Wuhan, China), rabbit-anti mouse VCAM1 (1:100, Cell Signaling Technology, Beverly, MA, USA). After incubation, the sections were rinsed in PBS and incubated with corresponding secondary antibodies containing Alexa Fluor 647 Donkey Anti-Goat IgG (Thermo fisher scientific MA, USA), Alexa Fluor 488 Donkey Anti-Mouse IgG (Thermo fisher scientific MA, USA), CY3-conjugated Donkey anti-rabbit IgG (Jackson Immuno-Research, West Grove, PA, USA) for 1 h at 25° C. Thereafter, sections were stained with 4,6-diamidino-2-phenylindole (DAPI) (10 μg/mL; Sigma-Aldrich) for 15 min at 25° C. To determine the percentage of neurons apoptosis, transferase-mediated nick end labeling (TUNEL) (Cell Death Detection Kit, Roche, Basel, Switzerland) and rabbit-anti NeuN (1:200; Abcam Shanghai, China) co-staining were performed. All sections were observed blindly under an Olympus BX51 fluorescent microscope (Olympus, Japan) or a laser scanning confocal microscope (FV500; Olympus, Tokyo, Japan). The parameters for image capture were set from a WT control group which were constant for all remaining images capturing.

For analysis, five fields of the hematoma boundary per section, four sections per animal (*n* = 4) were selected. The Iba-1(+) and MPO (+) cells were counted. The total number of TUNEL and NeuN double-positive cells in five fields near the injury area was counted with ImageJ (National Institute of Health, Bethesda, MD, USA).

### BBB permeability

The blood-brain barrier (BBB) permeability was measured by the extravasation of Evans Blue dye on days 1 and 3 post-ICH [22]. In brief, a 2% solution of Evans Blue in sterile saline (4 ml/kg of body weight, Servicebio, China) was injected into the tail vein 3 h before mice were sacrificed. Then, mice were transcardially perfused with 40 ml of 0.9% cold saline. Afterward, brain tissues were quickly removed, weighed, and homogenized in 1100 μl PBS. After sufficient grounding, the samples were centrifuged at 6000×*g* for 30 min. The supernatant was collected and mixed with an equal amount of 50% trichloroacetic acid (Sigma-Aldrich, St Louis, MO). Samples were incubated overnight at 4 °C and centrifuged for 30 min (6000×*g*, 4 °C). Evans Blue dye were measured by a spectrophotometer (Thermo Spectronic Genesys 10 UV, Thermo Fischer Scientific Inc., Waltham, MA, USA) at 610 nm and quantified from a standard curve. The results are presented as (microgram of Evans Blue dye)/(gram of tissue).

### Neurological outcomes

Behavioral assessments were done by researchers blinded to this experiment. Three rating systems were used to evaluate the neurological outcome from day 0 to day 10 after ICH as described previously [23]. The Longa score was rated on a 5-point scale (0, no apparent deficit; 1, slight instability while walking without circling; 2, circling toward the right with some straight movement; 3, heavy circling toward the right without straight movement or no movement at all; 4, deceased). Meanwhile, a standard forelimb placing test was carried to assess the sensory and motor impairment. In this experiment, each mouse was tested 10 times for each forelimb, and the percentage of trials in which the mouse placed the appropriate forelimb on the edge of the countertop in response to the vibrissae stimulation was determined. The third behavioral test involved a corner turn test. A mouse was allowed to proceed into a corner, the angle of which was 30°. To get out of the corner, the animal could turn either the left or right limb and the choice was recorded. This was repeated 10 times, and the percentage of right turns was calculated. Only turns involving full rearing along each wall were included.

### Western blot

Mice were anesthetized and perfused quickly with 0.9% sterile saline solution on days 1 and 3 after ICH (*n* = 5 for each time point). Tissue proteins from the perihematoma region were extracted and western blotting was performed. Total protein (40 μg) was loaded on 10% TGX Stain-Free FastCast gel electrophoresis (Bio-Rad, CA, USA). After electrophoresis, the proteins were transferred to a polyvinylidene fluoride (PVDF) membrane (0.45 μm; Millipore Corporation, Bedford, MA, USA). After blocking with 5% nonfat milk in tris buffer solution (TBS) (pH7.4) containing 0.1% Tween 20, the membranes were incubated overnight with antibodies including mouse anti-β-actin (1:1000; Promoter, Wuhan, China), rabbit anti-TREK-1 (1:500; Alomone Lab, Jerusalem, Israel), rabbit anti-ZO-1/occludin (1:500; Thermo fisher scientific MA, USA), mouse anti-claudin-5 (1:500; Thermo Fisher Scientific, MA, USA), rabbit anti-Aquaporin 4 (AQP4) (1:500, Proteintech Wuhan, China), and rabbit anti-MMP-9 (1:1000; Cell Signaling Technology, Beverly, MA, USA) diluted in blocking buffer overnight at 4 °C. After incubation with primary antibodies, the membranes were washed and transferred into a buffer with horseradish peroxidase (HRP)-conjugated immunoglobulin G (IgG) (1:6000; Boster, Wuhan, China) for 1 h at 25 °C. Finally, a Bio-Rad Chemi Doc XRS+ imaging system was used to obtain band imaging. The optical densities (OD) of signals were analyzed using ImageJ software. The integrated optical density (OD) of the signals was semi-quantified and expressed as the ratio of OD from the tested proteins to OD from the control of β-actin.

### Magnetic resonance imaging and measurement

Mice were deeply anesthetized throughout the MRI examination on days 1 and 3 post-ICH. MRI scanning was performed on a 3.0 T MR scanner (Discovery MR750 3.0 T, GE Medical Systems, LLC) using a mouse magnetic resonance coil (ChenGuang, Shanghai, China) at the Center for Imaging of Tongji Hospital. The imaging protocol for all mice included a T2 fast spin-echo and enhanced T2 star-weighted angiography (ESWAN) gradient-echo sequences. The field of view was 20 × 20 mm and the matrix was 256 × 256 mm. Seven coronal slices (1 mm thick) were acquired from the frontal pole to the brain stem and the images were preserved as 256 × 256 pixels pictures.

### ELISA

On days 1 and 3 after ICH, the perihematoma tissues (*n* = 4) were homogenized and centrifuged. The supernatants were collected and the concentrations of cytokines, including tumor necrosis factor-α (TNF-α), interleukin-1β (IL-1β) were measured using an enzyme-linked immunosorbent assay (ELISA) reagent kit (Dakewe Biotech Company, Shenzhen, China) following the manufacturer’s instructions. The concentrations of these factors were calculated based on a standard curve and expressed as g/mg.

### TEM imaging

To observe the ultrastructure of BBB, mice were deeply anesthetized and then transcardially perfused with 0.9% saline on days 1 and 3 after ICH. Afterward, the brains were quickly removed, cut into small parts (2 mm^3^) and further immersed in a fixative containing 2% paraformaldehyde and 2% glutaraldehyde at 4°C overnight. After washed thoroughly, the samples were post-fixed with 1% osmium tetroxidein at 4 °C for 2 h, and then blocked-stained with 2% aqueous solution of uranyl acetate for 1 h. Thereafter, the brain was dehydrated with graded ethanol and embedded in acrylic resin. Serial ultrathin (600 Å-thin) sections were cut and stained with 6% lead citrate and 3% uranyl acetate. The ultrastructure of BBB was observed using a transmission electron microscopy (TEM) (HT7700, Hitachi, Tokyo, Japan) blindly.

### Statistical analysis

The statistical analysis was performed using SPSS 20.0 software (IBM, Chicago, IL, USA). The differences between two groups were determined by the Mann-Whitney *U* two-tailed test. One-way or two-way analysis of variance (ANOVA) was used to compare the differences among multiple groups and Tukey’s post hoc tests were employed for the two groups’ comparison within the multiple groups. All data are expressed as mean ± SEM. The difference was considered significant at *p* < 0.05.

## Results

### Expression of TREK-1 in the BBB of normal brain and its dynamic variations after ICH

**Fig. 1:**
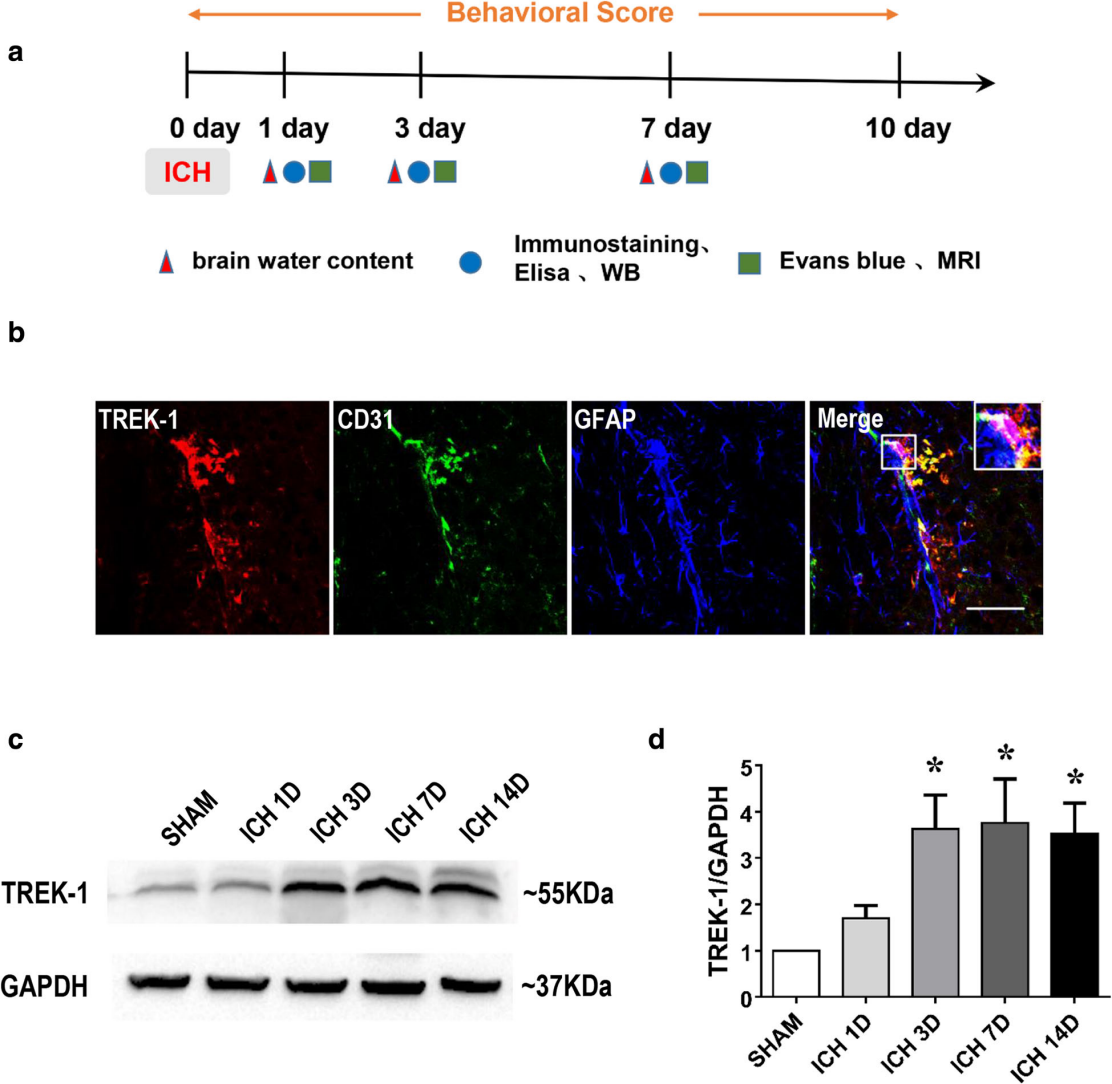
Expression of TREK-1 in the BBB of normal mice brain and perihematoma tissue after ICH. **a** The diagram of the experimental design in this study. **b** The localization of TREK-1 immunoreactivity was observed by immunofluorescent staining of TREK-1 (red) with GFAP (blue), and CD31 (green), respectively. Scale bar = 50 μm. **c** Representative western blot image of TREK-1 and GAPDH expression in perihematoma tissue. **d** Statistical analysis of western blots signals of TREK-1 in the perihematoma tissue of WT mice after ICH. The data were expressed as mean ± SEM and evaluated by one-way ANOVA with Tukey post hoc test (*n* = 5). *p < 0.05, sham group versus ICH 3d, 7d, and 14d groups

Figure 1a shows the diagram of the experimental design in this study. We examined the expression of TREK-1 in BBB at the caudate nucleus of WT mice using immunofluorescent staining. TREK-1 immunoreactivity was well co-localized with GFAP-positive astrocytes and CD31-positive endothelial (Fig. 1b). We then investigated the dynamic changes of TREK-1 protein expression after ICH using western blot. Western blot analysis revealed significant increases in the TREK-1 protein levels in the perihematoma tissue on days 3, 7, and 14 after ICH compared with sham control (*p* < 0.05) (Fig. 1c, d , Additional file 1: Figure S1).

### Deficiency of TREK-1 increases the hematoma volume, encephaledema formation and AQP4 expression after ICH

**Fig. 2:**
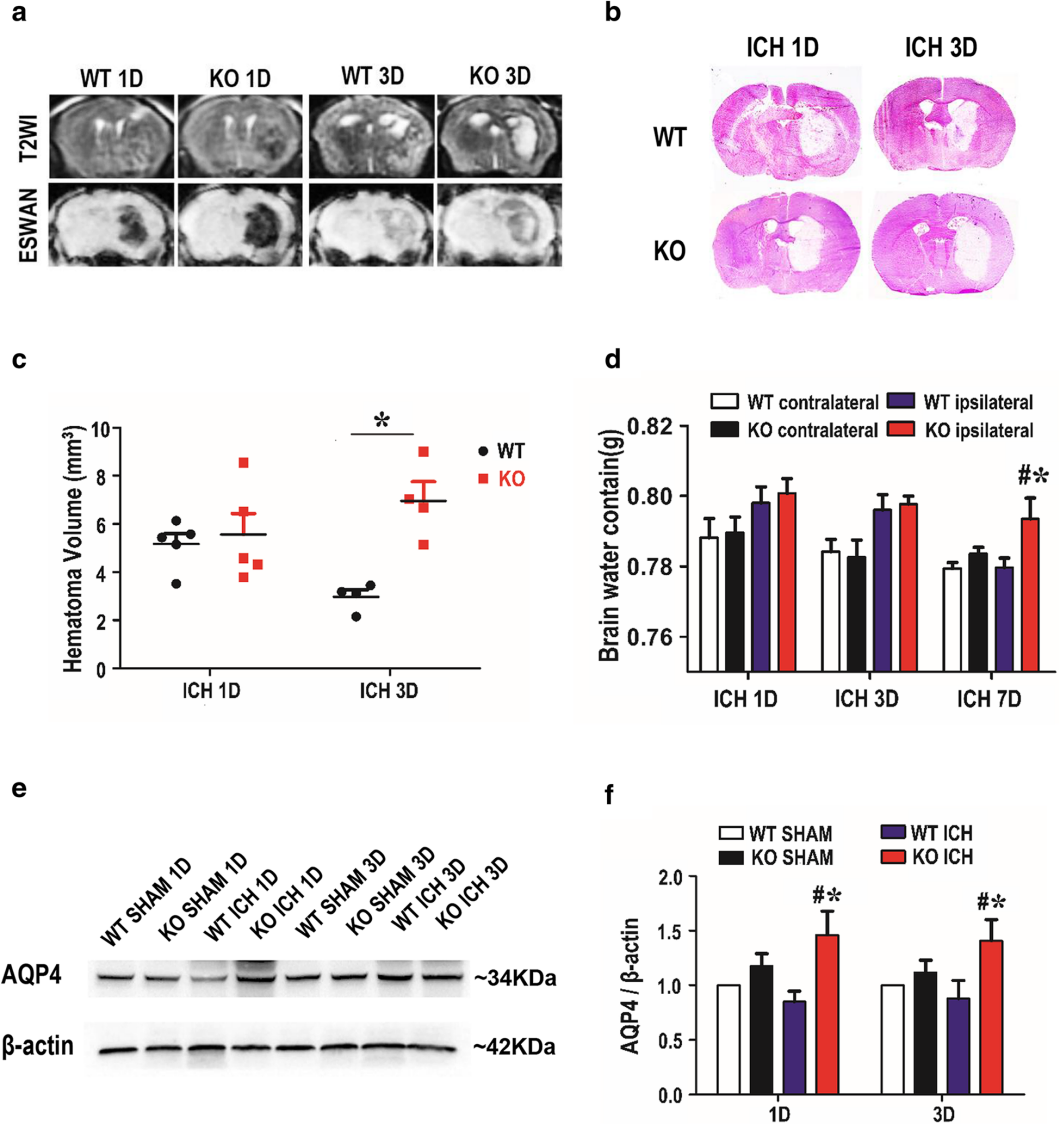
TREK-1-deficient mice possess a larger hematoma volume on day 3 after ICH and more brain water content on day 7 post-ICH. **a** A representative MRI image on days 1 and 3 after ICH. **b** Representative image of HE staining on days 1 and 3 after ICH. **c** Statistic analysis of hematoma volume according to the HE staining. **d** Statistical analysis of brain water content using the wet/dry weigh method on days 1, 3, and 7 after ICH. **e** Representative western blot image of AQP4 expression in the perihematoma tissue. **f** Quantification of AQP4 expression according to the β-actin expression on days 1 and 3 after ICH. Values were expressed as mean ± SEM and data were evaluated by one-way ANOVA with Tukey post hoc test (*n* = 5–7). ^#^*p* < 0.05, WT sham group versus KO ICH group, *p < 0.05, WT ICH group versus KO ICH group

Mice were subjected to MRI examination on days 1 and 3 after collagenase administration. MRI showed significant hematoma and brain edema on day 1 and 3 after ICH. On day 1 after ICH, T2-weighted MRI showed a mixed signal, whereas ESWAN images showed hypointense signal, suggestive of hematoma tissue. On day 3, the ESWAN images showed a mixed signal in the hematoma region whereas T2-weighted MRI showed a region with high signal intensity surrounded by dark rims, suggestive of perihematoma edema. The hematoma and edema observed in KO mice were larger than WT mice (Fig. 2a). To further investigate the effect of TREK-1 deficiency on hematoma volume, a series of cryosections were processed with HE staining and hematoma volume was quantified. On days 1 and 3 after ICH, the hematoma volume in TREK-1^−/−^ mice was larger than that in WT mice (*p* < 0.05) (Fig. 2b, c). We further examined brain edema using a wet/dry method on days 1, 3 and 7 after ICH. All ICH animals revealed a significant increase of brain water content ipsilateral brain compared with sham-operated. TREK-1^−/−^ mice presented significantly higher water content than that in WT group on day 7 post-ICH (*p* < 0.05) (Fig. 2d, Additional file 1: Figure S2a). AQP4 proteins are abundantly expressed in perivascular astrocytes end foot which has been revealed to play a key role in modulating brain water transport in perihematoma region after ICH [24]. We quantified the AQP4 protein expression by western blot analysis. In WT animals, the expression of AQP4 protein level did not change significantly after ICH. However, compared with WT ICH group, the AQP4 level was evidently upregulated in TREK-1 KO ICH group on days 1 day 3 after ICH (*p* < 0.05) (Fig. 2e, f, Additional file 1: Figure S2b,c).

### Deletion of TREK-1 exacerbates the blood-brain barrier impairment after ICH

**Fig. 3:**
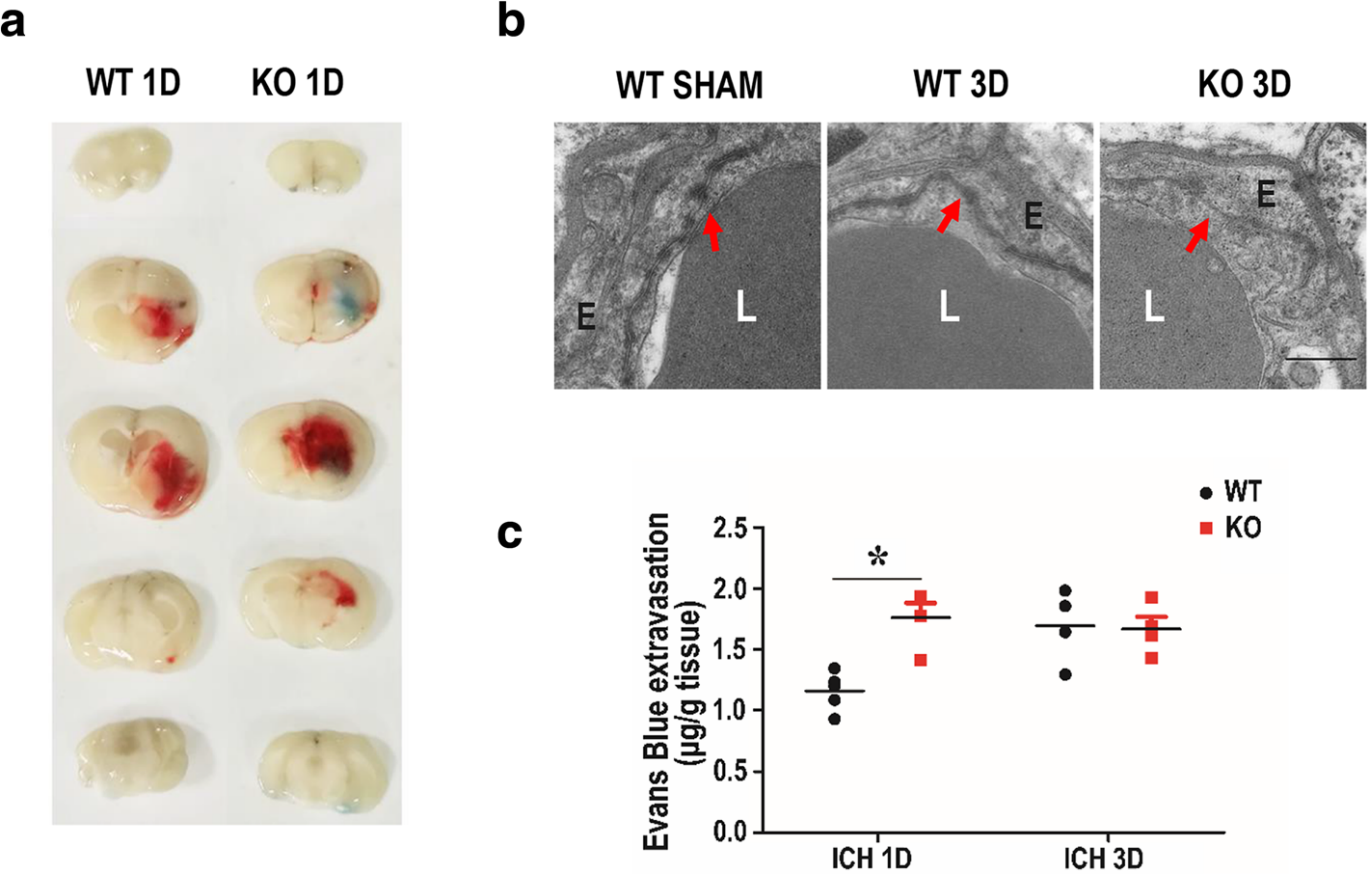
Deficiency of TREK-1 exacerbates the BBB impairment after ICH. **a** The representative brain slices show the extravasation of Evans Blue dye on day 1 after ICH. **b** The ultrastructure of the lumen (L), tight junctions (arrow) between endothelial cells (E) were observed under a TEM. **c** Statistic analysis of the extravasation of Evans Blue dye on days 1 and 3 after ICH. The values were represented as the mean ± SEM and data were evaluated by one-way ANOVA with Tukey post hoc test (*n* = 4–5). *p < 0.05, WT ICH group versus KO ICH group

Compromise of BBB often results in immune cell infiltration into the brain parenchyma, which is implicated in brain damage after ICH [25]. To test whether the deficiency of TREK-1 could mediate BBB breakdown, we injected Evans Blue dye through the tail vein and assessed the BBB integrity by assaying the leaked dye in the brain parenchyma. On day 1 after ICH, dye extravasation was observed in brain parenchyma of both WT and TREK-1 KO mice whereas there was more dye extravasation in TREK-1 KO animal than WT mice (*p* < 0.05) (Fig. 3a, c). Meanwhile, we observed the ultrastructure of BBB by TEM. In normal condition, the vascular walls were lined by regularly flattened endothelial cells and the intercellular cleft was sealed by tight junctions (arrow) that appeared as a series of electron-dense zones in TEM images. On day 3 after ICH, the tight-junctions of endothelial cells were disrupted appeared as reduced electron density and endothelium detachment. TREK-1 deficiency aggravates these ultrastructure damages of BBB after ICH compared with WT mice (Fig. 3b).

### Deficiency of TREK-1 promotes the MMP-9 expression while decreases the TJPs expression after ICH

**Fig. 4:**
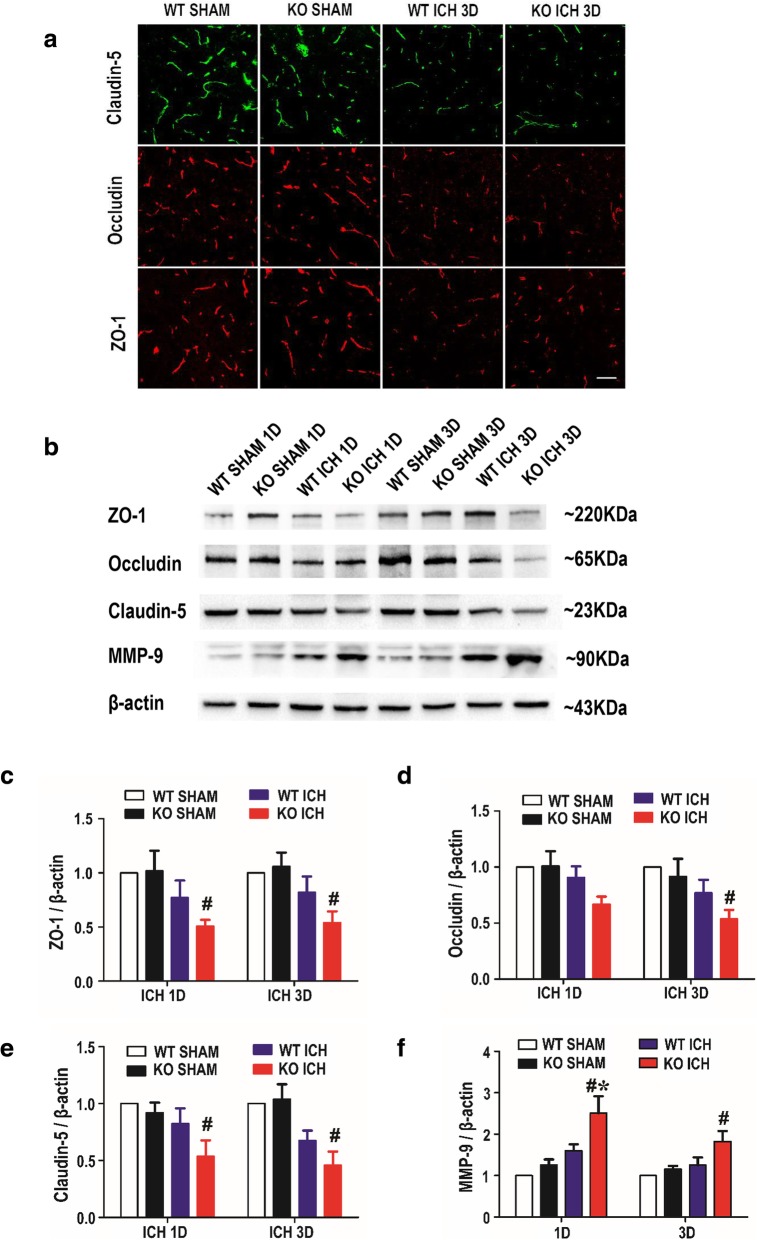
Deficiency of TREK-1 increases the MMP-9 protein expression but have no change on TJPs expression on days 1 and 3 after ICH compared with WT ICH group. **a** The immunofluorescent staining of the TJPs (ZO-1, occludin, claudin-5) on day 3 after ICH. Scale bar = 20 μm. **b** Representative western blots image of ZO-1, occludin, claudin-5, and MMP-9. **c**–**f** Quantifications of ZO-1, occludin, claudin-5, and MMP-9 protein levels in sham, ICH 1-day, and 3-day groups after ICH. All interest protein expression were normalized to β-actin. Values are expressed as mean ± SEM (*n* = 5), and data were evaluated by one-way ANOVA with Tukey post hoc test. ^#^*p* < 0.05, WT sham group versus KO ICH group, *p < 0.05, WT ICH group versus KO ICH group

Matrix metalloproteinases (MMPs) especially MMP-9 can cause an increase in capillary permeability, thereby contributing to BBB opening and brain edema after ICH [21, 26]. The MMP-9 protein expression was investigated using western blot analysis. On days 1 and 3 after ICH, MMP-9 expression was significantly increased in all animals compared with sham control. TREK-1 deficiency further promoted the MMP-9 expression compared with WT ICH mice (*p*<0.05) (Fig. 4b, f, Additional file 1: Figure S3a). Tight junctions are composed of transmembrane proteins include occludin, claudins, ZO-1, and the junctional adhesion molecules. Their loss and phosphorylation from the plasma membrane could result in BBB hyperpermeability and secondary brain damage after ICH [4]. We next detected TJPs expression using immunofluorescence staining and western blot. These results demonstrated that TJPs expression was markedly decreased on days 1 and 3 after ICH in TREK-1^−/−^ mice (*p* < 0.05) but not in WT ICH group (*p* > 0.05) in comparison with the sham group (Fig. 4a–e, Additional file 1: Figure S3b-d).

### Deficiency of TREK-1 aggravates microglia cell activation, neutrophil infiltration and promotes the secretion of pro-inflammatory cytokine IL-1β and TNF-α after ICH

**Fig. 5:**
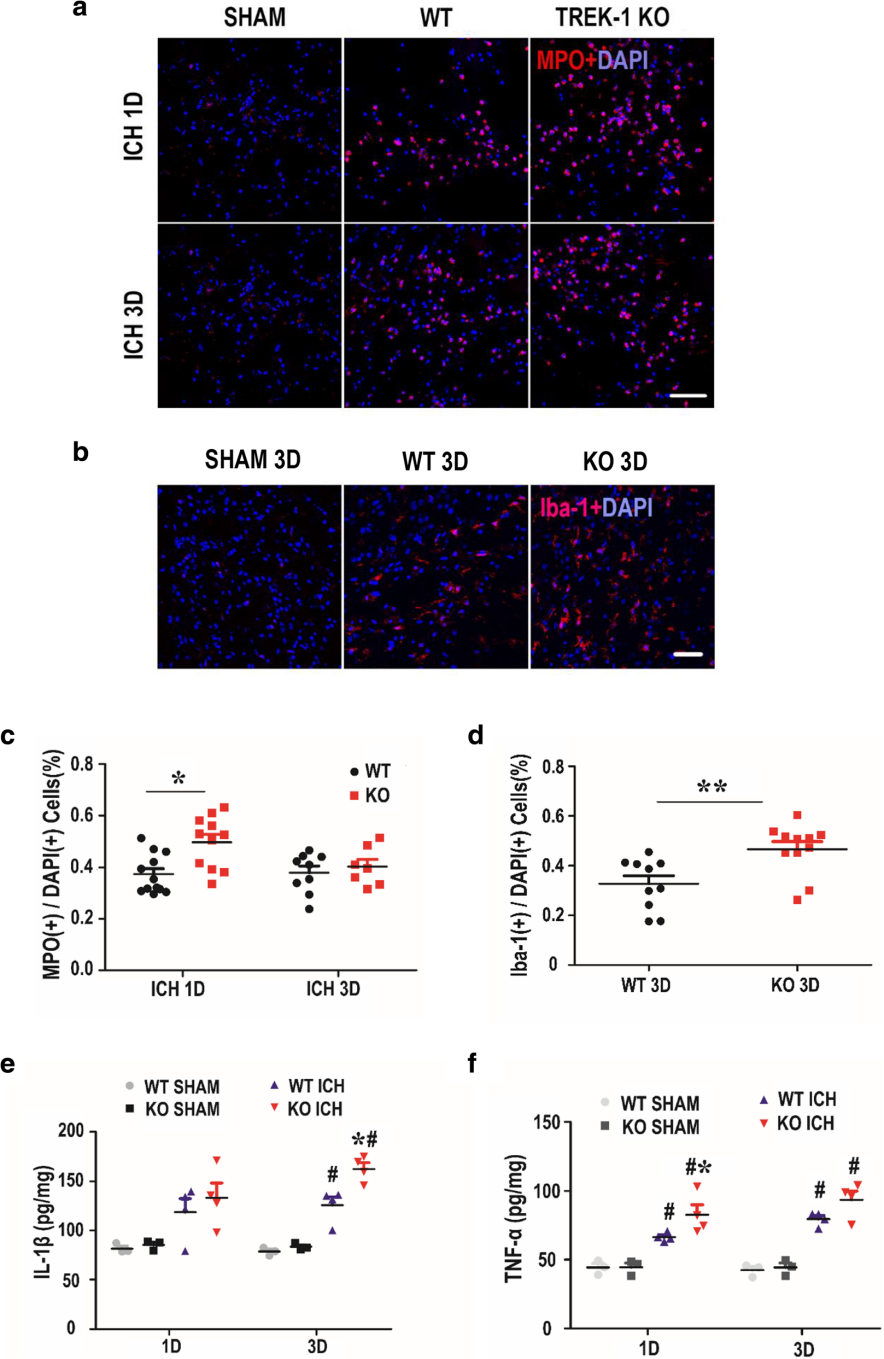
Deletion of TREK-1 aggravates microglia cell activation, neutrophil infiltration, and promotes the secretion of pro-inflammatory cytokine IL-1β and TNF-α on 1 day and 3 days after ICH. Immunofluorescent staining of MPO (**a**) and Iba-1 (**b**) with DAPI in the perihematoma region to detect activated microglia and infiltrated neutrophils on days 1 and 3 after ICH. Scale bar = 20 μm. **c**–**d** Statistical analysis of the percentage of activated microglia and infiltrated neutrophils. **e**–**f** ELISA result of TNF-α and IL-1β in the perihematoma zone on days 1 and 3 after ICH. Values are expressed as mean ± SEM (*n* = 5–7), and data were evaluated by Student’s independent sample *t* test and one-way ANOVA with Tukey post hoc test. *p < 0.05, **p < 0.01 WT ICH group versus KO ICH group, ^#^*p* < 0.05, WT SHAM group versus KO ICH group

Microglia activation and inflammatory response contribute to the evolution of secondary degeneration after ICH. On day 3 post-ICH, the activated microglia was evaluated by immunofluorescent staining of Iba1. After ICH, microglia were activated featured by hypertrophy of the cell body and increased Iba1 immunofluorescence intensity in perihematoma zone (Fig. 5b, Additional file 1: Figure S4a). TREK-1 KO mice showed higher Iba1 immunoreactivity than WT group on day 3 post-ICH (Fig. 5b, d) (*p* < 0.01). To examine the infiltration of systemic neutrophils, immunofluorescent staining of MPO was performed on days 1 and 3 after ICH. In contrast to sham animals only having few MPO positive (MPO^+^) cells (Fig. 5a, Additional file 1: Figure S4b), ICH animals showed a strong increase in the number of MPO^+^ cells. Moreover, TREK-1KO mice presented greater MPO expression in perihematoma region on day 1 post-ICH compared with WT ICH group (*p* < 0.05) (Fig. 5a, c). Pro-inflammatory cytokine IL-1β and TNF-α are the most important inflammatory mediators after ICH [27]. We detected the expression of IL-1β and TNF-α in the ipsilateral hemisphere at days 1 and 3 after ICH using ELISA. As indicated in Fig. 5e, f, on days 1 and 3 after ICH, the IL-1β and TNF-α expression increased apparently compared with sham control. TREK-1 deficiency significantly improved the IL-1β and TNF-α secretion after ICH compared with WT ICH group (*p* < 0.05).

### TREK-1 deficiency upregulates the protein expression of ICAM-1, VCAM-1, and PECAM-1 after ICH

**Fig. 6:**
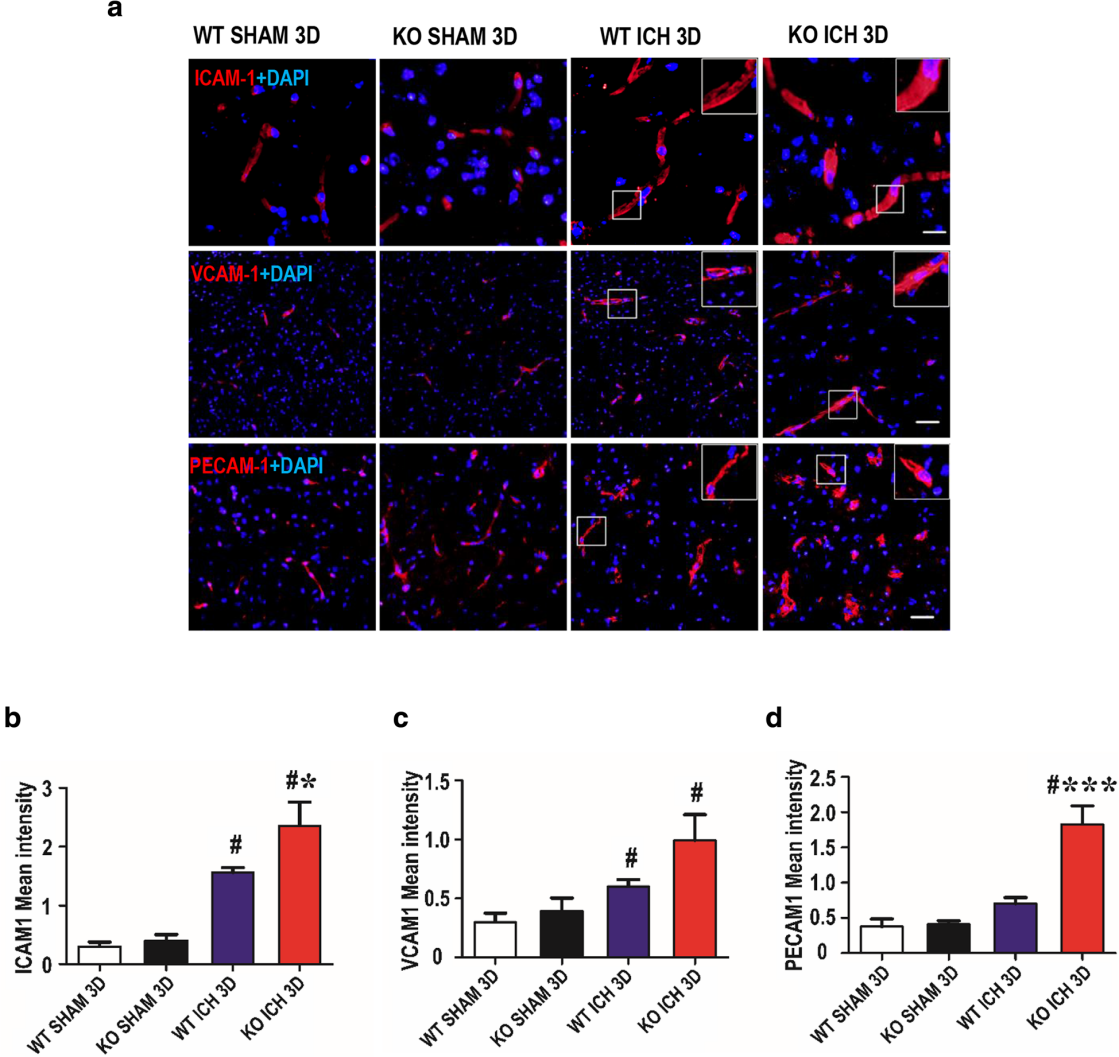
Deletion of TREK-1 promotes the ICAM-1, VCAM-1, and PECAM-1 protein expression after ICH. **a** Immunofluorescent staining of ICAM-1, VCAM-1, and PECAM-1 with DAPI in the perihematoma region on day 3 after ICH. Scale bar = 20 μm. **b**–**d** Semi-quantitative analysis of the mean immunofluorescent intensity of ICAM-1, VCAM-1, and PECAM-1. The data were represented as the mean ± SEM and evaluated by one-way ANOVA with Tukey post hoc test (*n* = 5–7). *p < 0.05, ***p < 0.001, WT ICH group versus KO ICH group. ^#^*p* < 0.05, WT sham group versus KO ICH group

The intercellular adhesion molecule 1 (ICAM-1), vascular cell adhesion molecule 1 (VCAM-1), and platelet endothelial cell adhesion molecule 1 (PECAM-1) has been reported to participate in BBB disruption and immune cell migration [18]. In this study, immunofluorescence staining was performed to investigate the expression of ICAM-1, VCAM-1, and PECAM-1 in the perihematoma region after ICH. On day 3 after ICH, expressions of these cell adhesion molecules (CAMs) were significantly upregulated in all animals compared with the sham group (*p* < 0.001). Meanwhile, the expression levels of ICAM-1and PECAM-1 in TREK-1 KO ICH group were much higher than those in WT ICH group (*p* < 0.05, *p* < 0.001) (Fig. 6a, b, d). TREK-1 deficiency did not induce a significant change in VCAM-1 expression after ICH compared with WT group (*p* > 0.05) (Fig. 6a, c).

### Deletion of TREK-1 increases the death of neurons and inhibits functional recovery after ICH

**Fig. 7:**
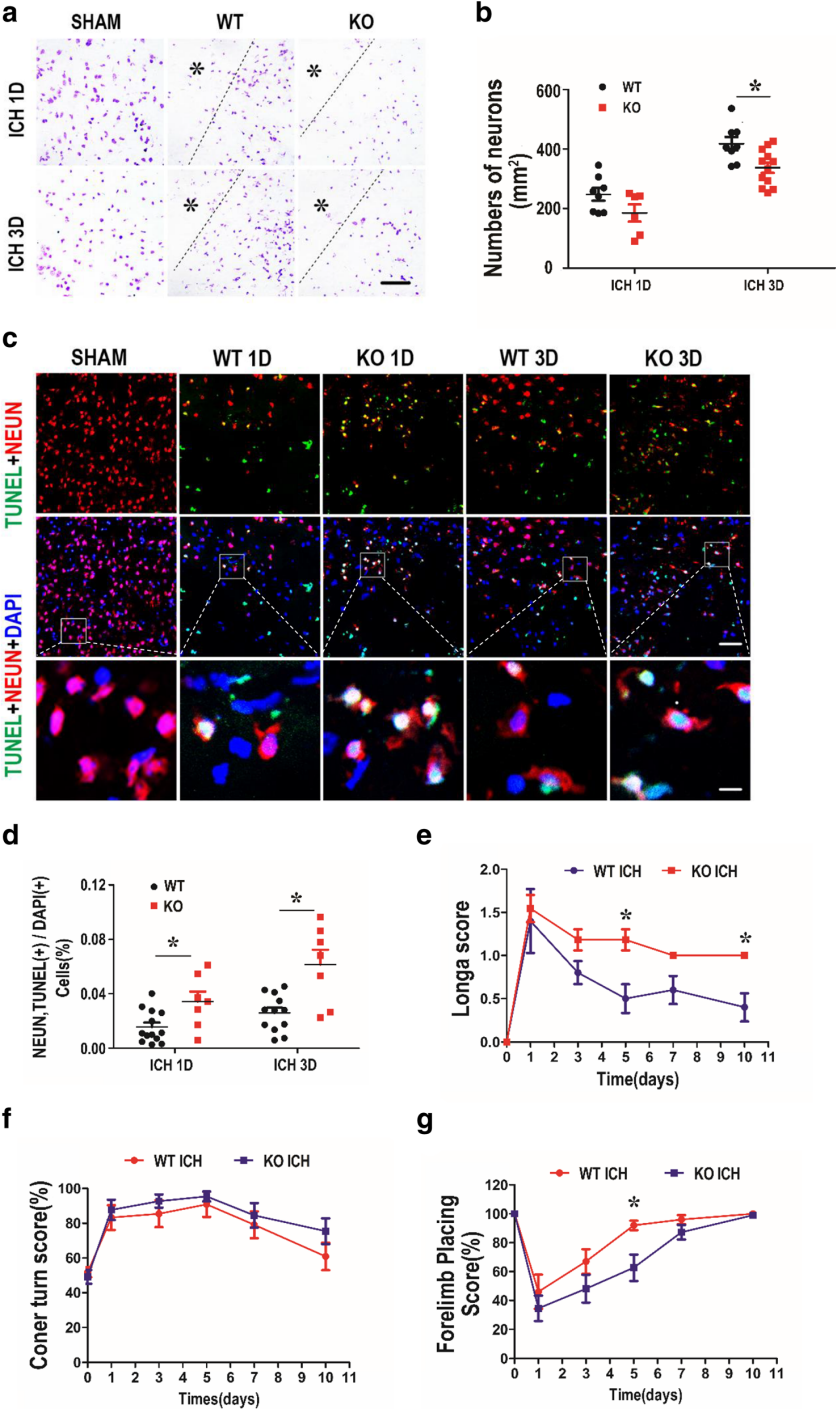
Deletion of TREK-1 increases the necrosis and apoptotic of neurons accompanied by retarded functional recovery after ICH. **a** A representative Nissl staining picture shows that TREK-1 deficiency exacerbated the necrosis of neurons. Scale bar = 50 μm. **b** Counting of Nissl-stained neurons in the perihematoma region on days 1 and 3 after ICH. **c** Apoptotic neurons in the perihematoma region on days 1 and 3 were detected by double staining of TUNEL (green), NeuN (red), and DAPI (blue). Scale bar = 50 μm. **d** Statistical analysis of percentage of apoptotic neurons on days 1 and 3 post-ICH in WT ICH and TREK-1 KO ICH groups. e–g Statistical analysis of Longa scores (**e**), corner turn scores (**f**), and forelimb placing scores (**g**) in WT and TREK-1 KO groups. Values are expressed as mean ± SEM (*n* = 10), and data were evaluated by Student’s independent sample *t* test. *p < 0.05, WT ICH group versus KO ICH group

On days 1 and 3 after ICH, the brain sections from ICH mice were stained with Nissl and TUNEL assay to determine cell death. Nissl staining showed that neuronal loss was observed after ICH which was significantly aggravated in TREK-1 deficiency mice compared with WT ICH group on day 3 after ICH (*p* < 0.05) (Fig. 7a, b). Subsequently, we performed TUNEL assay to investigate the effects of TREK-1 deletion on neuronal apoptosis. In the sham group, TUNEL-positive cells were rarely observed. These positive cells increased significantly in hematoma site after ICH on days 1 and 3 in both groups (Fig. 7c, Additional file 1: Figure S4c). Quantization of TUNEL^+^ cells in the perihematomal area indicates increased neuronal apoptosis in TREK-1 deficiency mice compared with WT ICH group (*p* < 0.05) (Fig. 7d). We next performed a series of neurological tests to assess neurological impairment and recovery after ICH [23, 28]. Neurological deficits were evident in all ICH-animals at 1 day after ICH as tested by the Longa score, the forelimb placing test, and the corner turn test. TREK-1 deficiency mice acquired worse Longa score and forelimb placing test score compared to WT mice over the following 10 days, suggesting the retarded functional recovery (*p* < 0.05) (Fig. 7e, g). But there was no significant difference in corner turn test score between WT ICH and TREK-1 KO ICH group (*p* > 0.05) (Fig. 7f).

## Discussion

TREK-1 is a member of newly discovered two-pore-domain background potassium channels which contribute to the background leak K+ currents and regulate neuronal excitability [7, 29]. Previous studies have found that TREK-1 exert active roles in depression, pain, cerebral ischemia, and spinal cord injury [12, 14, 30]. Bittner et al. have shown TREK-1 participate in BBB disruption-mediated inflammatory infiltration in a mice EAE model [18]. To date, its role in BBB disruption and inflammatory infiltration-related secondary neurologic impairment after ICH is unclear. Our study for the first time demonstrated that TREK-1 deficiency was implicated in BBB dysfunction and neuroinflammation following ICH in vivo. TREK-1 deficiency can aggravate BBB breakdown, inflammatory cells infiltration, neurons necrosis/apoptosis, and retard neurological functional recovery. The mechanism underlying TREK-1 deficiency on BBB compromise after ICH may be mediated by the upregulation of CAMs. Secondary injury after cerebral hemorrhage is the leading cause of neurological impairment and poor prognosis. Cerebral edema is a significant hallmark of secondary injury and also the main predictor of neurological outcome after ICH. The secretion of proinflammatory agent and the opening of the BBB contribute to progressive vasogenic edema [31]. BBB is the communication interface between the peripheral circulation and the CNS which is essential for the homeostasis and normal function of the brain [32, 33]. BBB disruption is a pathological hallmark of ICH-induced secondary injury. Such disruption contributes to the influx of immunocyte and edema formation. A range of factors has been implicated in BBB disruption after ICH, including inflammatory mediators, thrombin, hemoglobin breakdown products, oxidative stress, complement, and matrix metalloproteinases [3, 34]. Our data demonstrate that TREK-1 deficiency aggravated the post-ICH cerebral edema, exacerbated the disruption of BBB, and promoted the development of consequent neurodegeneration after ICH.

Increasing evidence indicates that inflammatory mechanisms have participated in the progression of ICH-induced secondary brain injury. Inflammatory responses post-ICH include the activation of microglia, infiltration of peripheral neutrophils, or macrophages and release of cytokines, chemokines, protease, and oxygen free radicals [35, 36]. Microglia and macrophages produce pro-inflammatory cytokines including TNF-α and IL-1β exerting a deleterious effect on perihematoma tissue after ICH [27]. Systemic immune cells, specifically blood-derived leukocytes, accumulating at the hematoma site and producing pro-inflammatory cytokines/MMPs are the principal orchestrators of the BBB damage following ICH [35, 37]. Recent evidence shows that TREK-1 plays a significant role in regulating immune response and BBB function in an EAE animal model [18]. We have previously identified that TREK-1 activator linolenic acid could inhibit microglia activation after brain ischemia [16]. Deficiency of TREK-1 significantly exacerbated focal inflammatory responses after spinal cord injury [14]. In this study, we found that deletion of the TREK-1 gene significantly exacerbated inflammatory reaction post-ICH as indicated by the increased microglia activation, the neutrophil influx, IL-1β /TNF-α secretion, and MMP-9 expression. TREK-1 has been rarely detected in microglia in previous studies [16, 38], suggesting that the increase of microgliosis may be indirectly mediated by TREK-1 deficiency induced BBB dysfunction and neuronal death.

AQP4 is the predominant water channel in the CNS and distinctively expressed in astrocyte processes and the basolateral cell plasma membrane of ependymal cells [39]. AQP4 channels have been reported to participate in the pathologic cerebral edema formation and potassium spatial buffering [40]. In mice ICH model, AQP4 protein deficiency could lead to aggravated cerebral edema and increasing apoptosis after ICH [41, 42]. In our research, we found that deficiency of TREK-1 significantly increases the AQP4 protein expression on days 1 and 3 after ICH compared with WT mice. However, the brain water content of the KO ICH group showed a significant increase on day 7 but not on day 1 and 3. This discrepancy indicated that AQP4 might not be the primary mechanism of TREK-1 knockout-mediated brain edema after ICH.

ICAM-1, VCAM-1, and PECAM-1 are cell adhesion molecules that mediate interaction between immunocytes and endothelial cells to form a “docked” structure rich in F-actin during the inflammatory infiltration process. They are low expressed mainly by endothelial cells under normal condition. In the inflammatory process, the expression of these adhesion molecules can be upregulated to promote leukocyte adhesion and blood-derived monocytes transmigration [43]. The upregulation of these molecules has been identified to participate in numerous neurological disorders [44–46]. The membrane localization of several adhesion receptors, such as ICAMs, CD43, and CD44, were regulated by cytoskeleton through one or more members of the ezrin, radixin, and moesin (ERM) family of proteins [47]. The TREK-1 channel was previously found to co-localize with ezrin and induce the formation of actin-rich protrusions [48]. Bittner et al. have found these adhesion molecules play a role in the TREK-1 deficiency-mediated immune infiltration in mice EAE model [18]. In this study, we test these CAMs expression after ICH using immunofluorescent staining. Deficiency of TREK-1 channels promoted their expression on day 3 after ICH. Therefore, the adhesion molecule (CAMs)-mediated signaling pathway (such as F-actin cytoskeleton rearrangement) may constitute the mechanism underlying the TREK-1 deletion-mediated increased infiltration of inflammatory cells.

In the perihematomal tissue, neuronal death continues for 2–3 days and the death of which are the main criminal of functional impairment after ICH [49, 50]. This irreversible secondary neurodegeneration is thought to result from apoptosis or necrosis, due to toxic factors released from the hematoma zone such as reactive oxygen species, glutamate, hemoglobin, and its oxidized product hemin [51, 52]. Recent studies have revealed that TREK-1 inhibition aggravates neuronal apoptosis after focal ischemia and spinal cord injury [14, 16]. In this research, our data demonstrated that TREK-1 deficiency promoted neuronal necrosis and apoptosis after ICH. Moreover, these cellular degenerations were ultimately translated into considerable behavioral impairment. The neuronal destructive effects of TREK-1 deficiency may be mediated by multiple mechanisms including the direct apoptotic effect of TREK-1 inhibition and the indirect effect of TREK-1 deficiency-induced microenvironment of neuronal damage and repair including the inflammatory response, BBB compromise, and astrocyte dysfunction [14, 38].

TREK-1 channels are extensively expressed in neurons, astrocytes, and endothelial cells in CNS. Genetic deficiency of TREK-1 channels leads to increased BBB disruption, focal inflammatory, and neuronal death. The aggravated secondary damage in TREK-1^−/−^ mice may be attributed to the increased expression of CAMs, AQP4, MMP-9.These multiple players might promote the cerebral edema and exacerbated neurologic impairment post-ICH (The cover image in this issue in Additional file 1: Figure S4d).

## Conclusions

We described for the first time that TREK-1 deficiency plays detrimental roles in the secondary BBB injury and neuroinflammation after experimental mice ICH model. Deletion of TREK-1 could compromise BBB function, aggravate the inflammatory cascade, neuronal apoptosis, and inhibit neurologic functional restoration after ICH. Our research here suggest that TREK-1 may thus constitute a promising therapeutic approach for the treatment of secondary damage after ICH.

## Additional file

Additional file 1: **Figure S1.** (a) Immunofluorescent-stained images of the TREK-1 negative control co-stained with GFAP and DAPI. (b) The expression of TREK-1 in neurons was detected using immunofluorescent staining. (c-d) PCR and WB genotyping results of WT mice and TREK-1^−/−^ mice. (e-f) The full image containing the target band (TREK-1, GAPDH) with molecular weight markers. **Figure S2.** (a) Statistical analysis of ipsilateral brain water content in the ICH group compared to the sham group. (b-c) The full images containing the target band (AQP4, β-actin) with molecular weight markers. **Figure S3.** The full images containing the bands MMP9 (a), claudin-5 (b), occluding (c), ZO-1 (d) with molecular weight markers. **Figure S4.** (a) Co-staining of MPO negative control with DAPI. (b) Immunofluorescence staining of Iba-1 negative control with DAPI. (c) Immunofluorescent staining of TUNEL negative control co-stained with NeuN and DAPI.Scale bar = 50μm. (d) Cover image for this issue. **Table S1.** A detailed list of the mice used in different groups in this study. **Table S2.** A detailed catalog of antibodies and reagents used in this study including the manufacturer, working concentration, and the catalog number. (PDF 838 kb)

## Abbreviations

AQP4: Aquaporin4 ANOVA: Analysis of variance
BBB: Blood-brain barrier
CAMs: Cell adhesion molecules
CNS: Central nervous system
EAE: Experimental autoimmune encephalomyelitis
GFAP: Glial fibrillary acidic protein
IL-1β: Interleukin-1 beta
ICH: Intracerebral hemorrhage
ICAM-1: Intercellular adhesion molecules1
KO: Knockout
MRI: Magnetic resonance imaging
MMP-9: Matrix metalloproteinase-9
PBS: Phosphate buffer solution
PECAM-1: Platelet endothelial cell adhesion molecule1
SEM: Standard error of the mean
TNF-α: Tumor necrosis factor alpha
TREK-1: TWIK-related K^+^ channel 1
TBS: Tris-buffered saline
TUNEL: Terminal-deoxynucleotidyl transferase-mediated nick end labeling
TJPs: Tight junction proteins
K2P: Two-pore domain potassium channels
TEM: Transmission electron microscopy
VCAM-1: Vascular cell adhesion molecule1
