# Supplementary material for: Deficiency of TREK-1 potassium channel exacerbates blood-brain barrier damage and neuroinflammation after intracerebral hemorrhage in mice
Source: J Neuroinflammation. 2019 May 9;16:96. doi: 10.1186/s12974-019-1485-5 (PMC6506965; doi:10.1186/s12974-019-1485-5)
Supplement: Supplementary file 1 — Figure S1. (a) Immunofluorescent-stained images of the TREK-1 negative control co-stained with GFAP and DAPI. (b) The expression of TREK-1 in neurons was detected using immunofluorescent staining. (c-d) PCR and WB genotyping results of WT mice and TREK-1−/− mice. (e-f) The full image containing the target band (TREK-1, GAPDH) with molecular weight markers. Figure S2. (a) Statistical analysis of ipsilateral brain water content in the ICH group compared to the sham group. (b-c) The full images containing the target band (AQP4, β-actin) with molecular weight markers. Figure S3. The full images containing the bands MMP9 (a), claudin-5 (b), occluding (c), ZO-1 (d) with molecular weight markers. Figure S4. (a) Co-staining of MPO negative control with DAPI. (b) Immunofluorescence staining of Iba-1 negative control with DAPI. (c) Immunofluorescent staining of TUNEL negative control co-stained with NeuN and DAPI.Scale bar = 50μm. (d) Cover image for this issue. Table S1. A detailed list of the mice used in different groups in this study. Table S2. A detailed catalog of antibodies and reagents used in this study including the manufacturer, working concentration, and the catalog number. (PDF 838 kb) [file 12974_2019_1485_MOESM1_ESM.pdf]

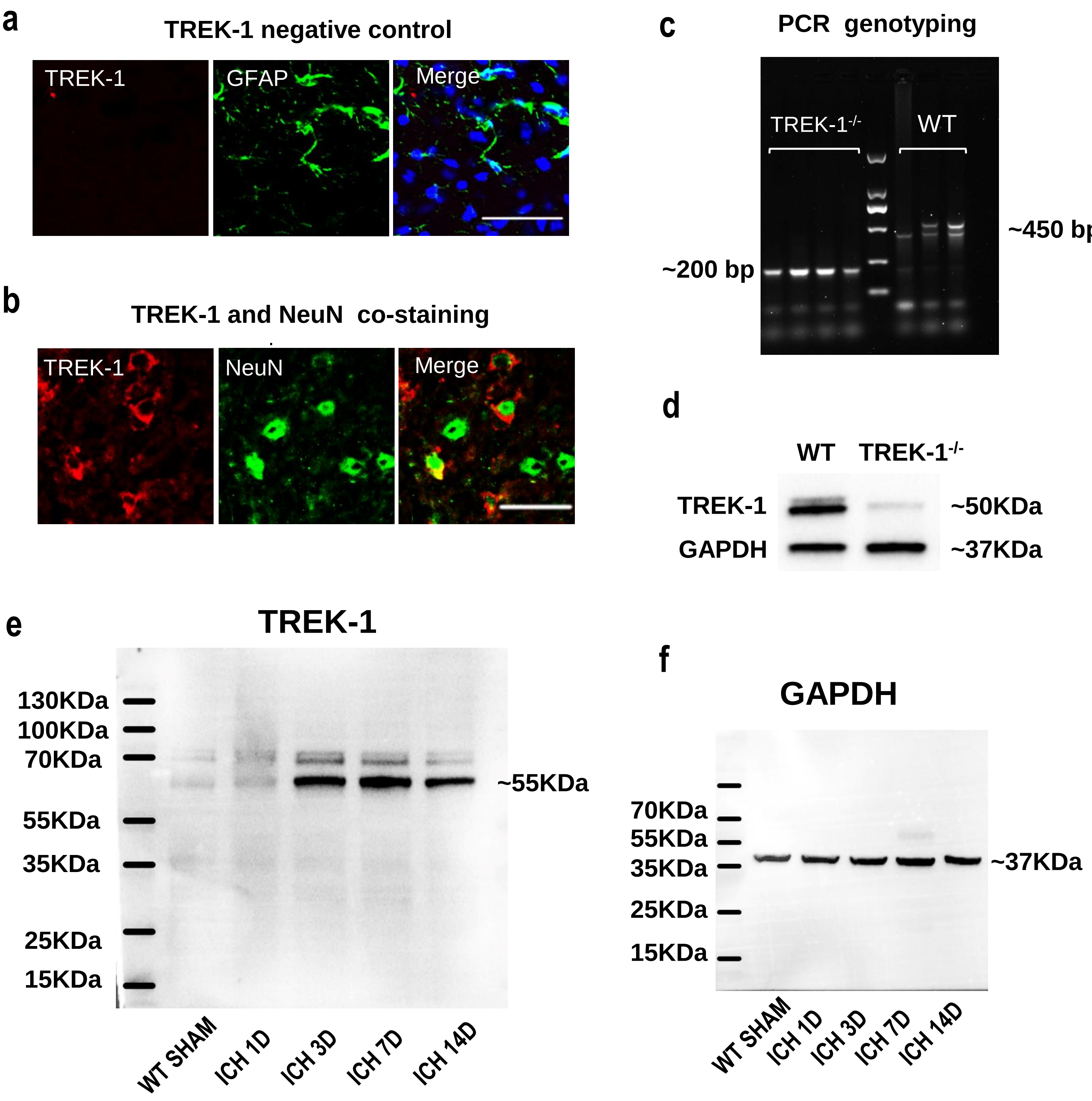

Supplementary Figure 1

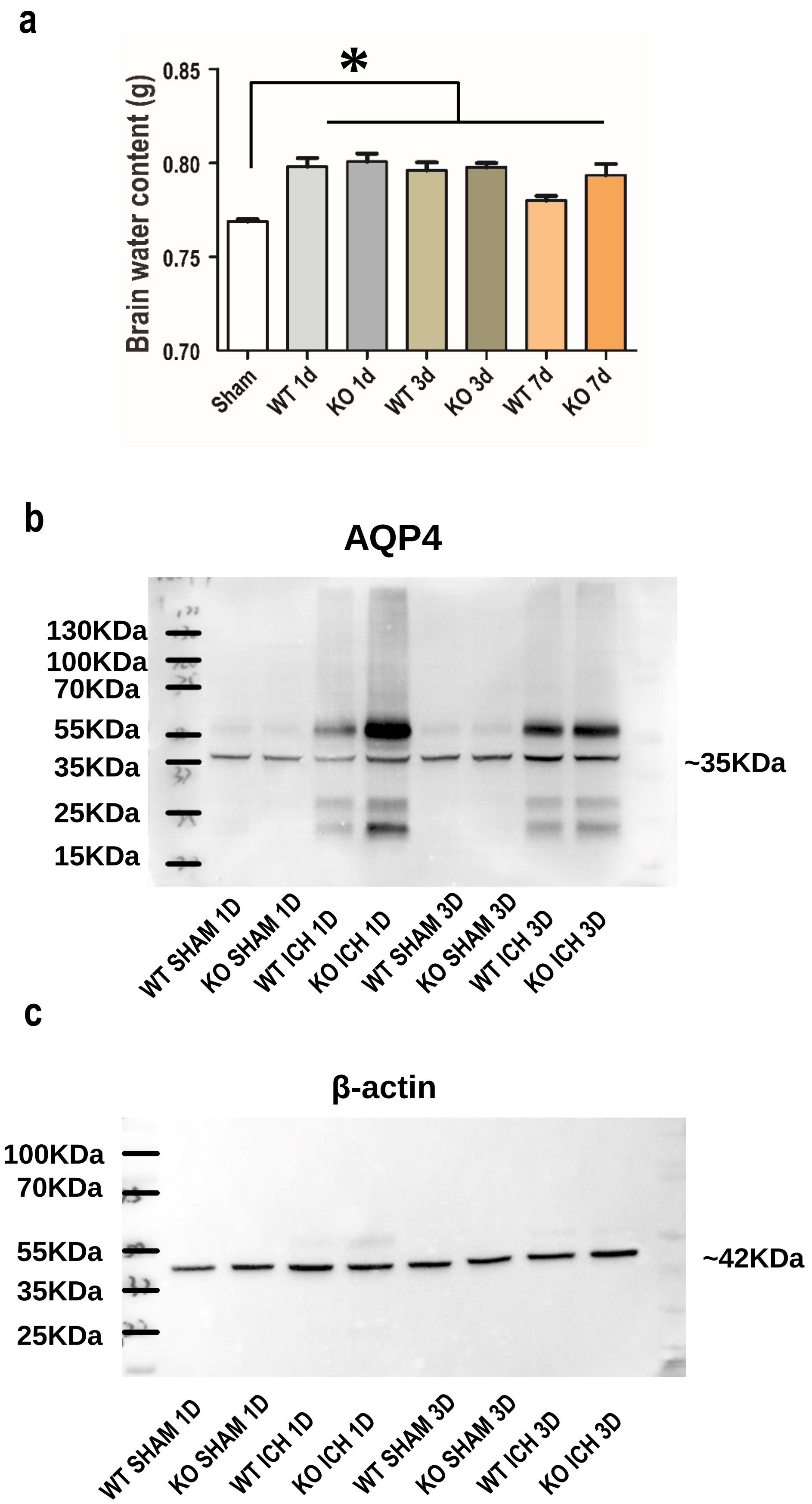

Supplementary Figure 2

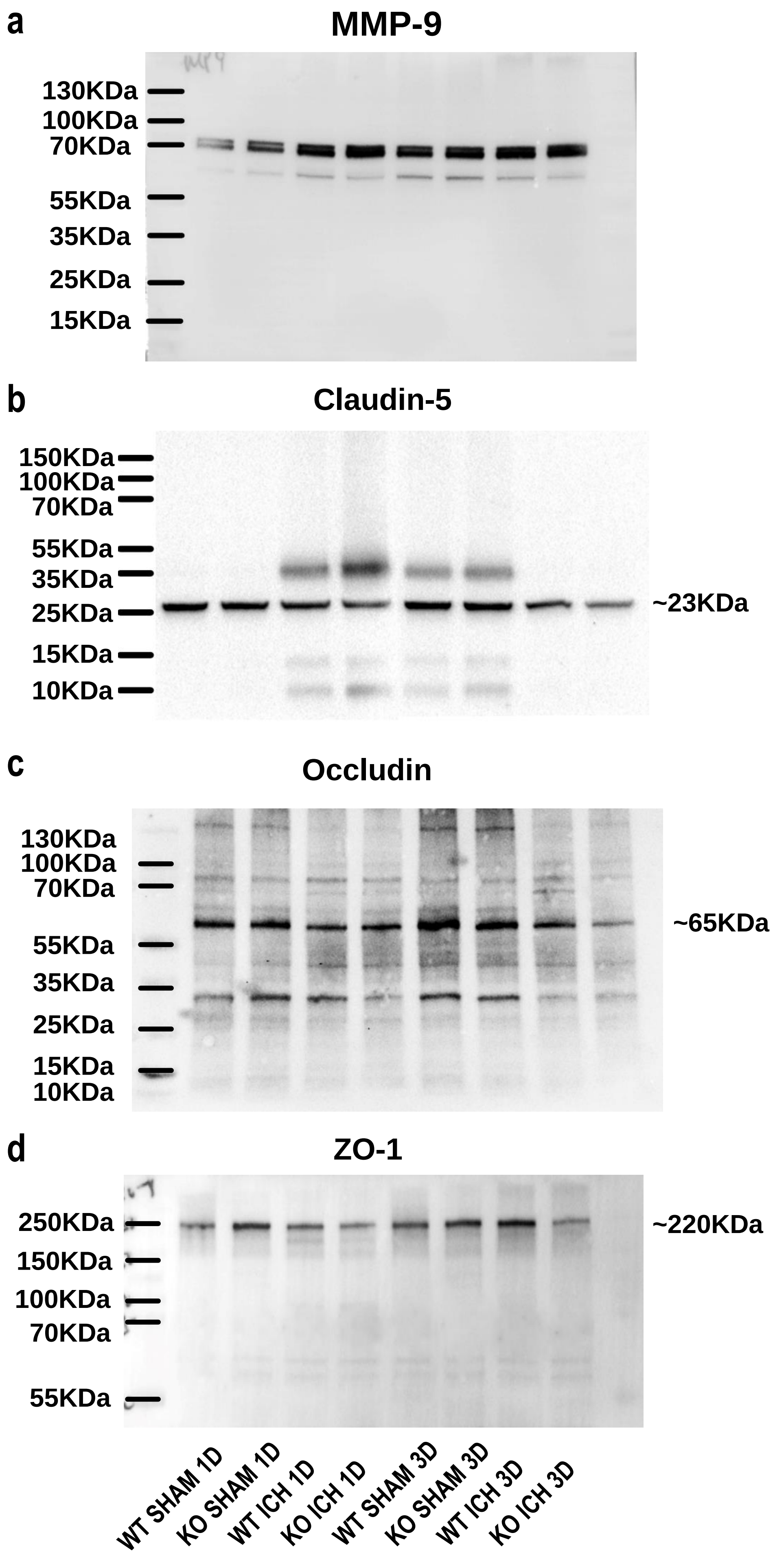

Supplementary Figure 3

Negative control

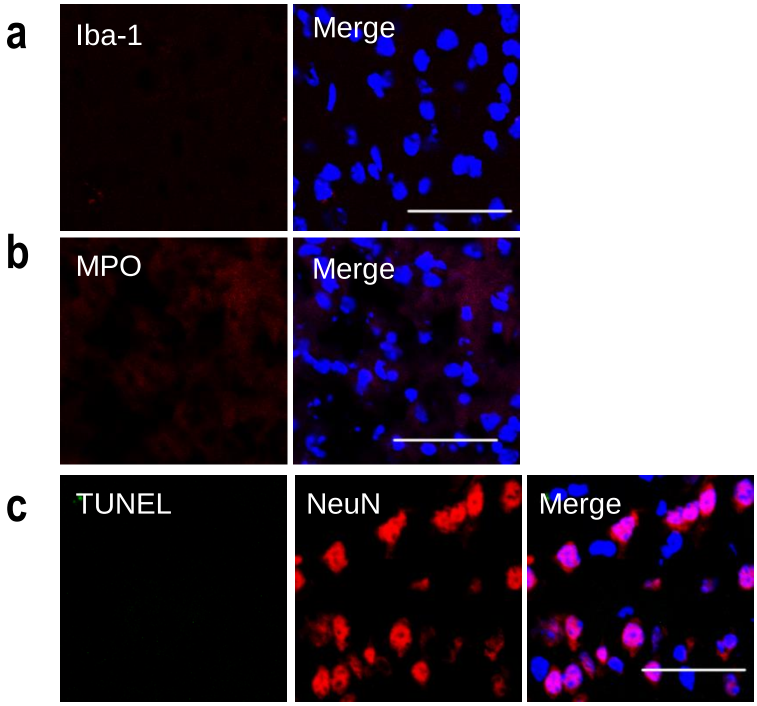

d Cover image for this issue

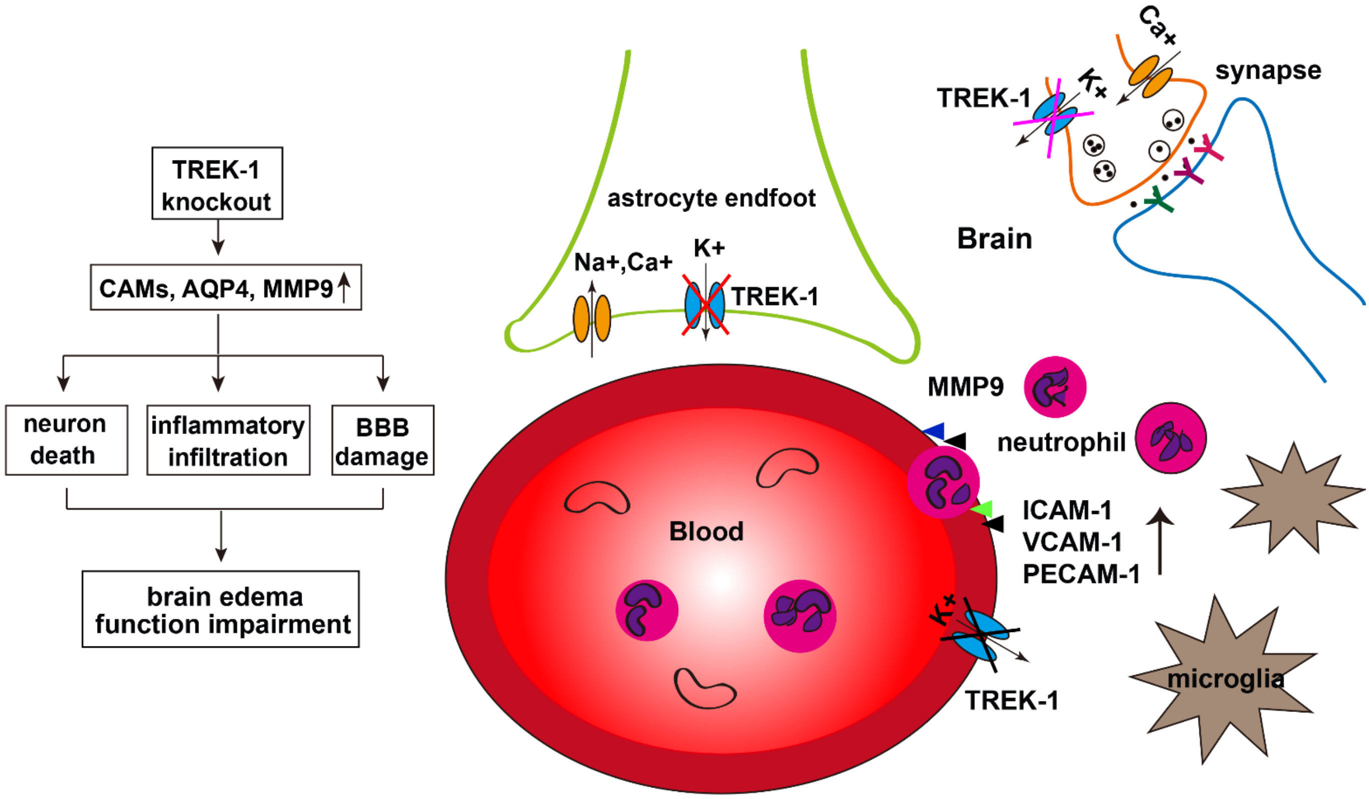

Supplementary Figure 5

Study Design

Total 213 mice

Time points / experiments	WT sham	KO sham	WT ICH 1D	KO ICH 1D	WT ICH 3D	KO ICH 3D	WT ICH 7D	KO ICH 7D
behavior	3	3	10	10				
Brain water content	–	–	7	7	7	7	5	5
Evans blue extravasation	3	3	5	4	3	3	–	–
immunofluorescent staining	3	3	5	5	5	5	–	–
Western blotting	5	5	5	5	5	5	–	–
ELISA analysis	4	4	4	4	4	4	–	–
hematoma assessment	–	–	5	5	5	5	–	–
MRI	–	–	3	3	3	3	–	–
TEM	2	2	3	3	3	3	–	–

Supplementary Table 1

Antibodies Catalog

Reagent	Manufacturer	concentration	Catalog number
**TREK-1**	Alomone Lab, Jerusalem, Israel	1:100(IF)<br>1 : 500(WB)	APC-047
**Iba-1**	Wako, Osaka, Japan	1 : 200(IF)	019-19741
**MPO**	Santa Cruz Biotechnology ,Texas, USA	1:100(IF)	SC-16128-R
**GFAP**	Abcam Shanghai, China	1:200(IF)	ab-53554
**CD31**	BD Bioscience New Jersey ,USA	1:100(IF)	550274
**Claudin-5**	Thermo fisher scientific Massachusetts, USA	1:200(IF)<br>1:700(WB)	35-2500
**Occludin**	Thermo fisher scientific Massachusetts, USA	1:200(IF)<br>1:700(WB)	71-1500
**ZO-1**	Thermo fisher scientific Massachusetts, USA	1:200(IF)<br>1:700(WB)	61-7300
**AQP4**	proteintech Wuhan, China	1:50(IF)	16473-1-AP
**MMP9**	Cell Signaling Technology, Beverly, MA, USA	1:1000(WB)	13667
**ICAM1**	proteintech Wuhan, China	1:100(IF)	10020-1-AP
**VCAM1**	Cell Signaling Technology, Beverly, MA, USA	1:100(IF)	39036
**β-actin**	Boster ,Wuhan, China	1:1000(WB)	BM0627
**TUNEL apoptosis Detection Kit**	Roche, Basel, Switzerland		11684795910
**Donkey anti-Mouse IgG Alexa Fluor 488**	Thermo fisher scientific Massachusetts, USA	1:300	A-21202
**CY3-conjugated goat anti-rabbit IgG**	Jackson Immuno-Research, West Grove, PA, USA	1:300	111-166-003
**horseradish peroxidase (HRP) conjugated goat anti-rabbit IgG**	Boster, Wuhan, China	1 : 6000	16-04-26
**HRP-conjugated goat anti-mouse IgG**	Boster, Wuhan, China	1 : 6000	16-04-29
**IL-1 Elisa kit**	Neobioscience,Shenzhen ,China		M170718-001a
**TNF-α Elisa kit**	Neobioscience,Shenzhen ,China		M170718-102a

Supplementary Table 2
